# N,S-doped carbon dots derived from tryptophan for sensitive and selective detection of meropenem in live cells, human urine, and water samples

**DOI:** 10.3389/fchem.2026.1924756

**Published:** 2026-10-27

**Authors:** Xincheng Hu, Yuerong Zou, Kanagaraj Thangamani, Rangasamy Kayalvizhi, Arivazhagan Anandhan, Tamilselvi Damodaran, Kanagaraj Rajalakshmi, Dongwei Zhu, Selvaraj Muthusamy, Yanhong Chen

**Affiliations:** 1 Department of Immunology and Laboratory Medicine, Jiangsu Key Laboratory of Laboratory Medicine, School of Clinical Medicine & Laboratory Medicine, Jiangsu University, Zhenjiang, China; 2 Department of Chemistry, Sri Eshwar College of Engineering, Coimbatore, Tamil Nadu, India; 3 Department of Chemistry, Erode Sengunthar Engineering College, Erode, Tamil Nadu, India; 4 Department of Chemistry, Rathinam Technical Campus, Coimbatore, Tamil Nadu, India; 5 Department of Clinical Laboratory, Xuzhou Central Hospital, Southeast University, Xuzhou, Jiangsu, China

**Keywords:** biological samples, carbapenem, fluorescence, meropenem, N,S-doped carbon dots, tryptophan

## Abstract

Meropenem (MP) is an important carbapenem antibiotic used as a last-line treatment that requires precise quantification for therapeutic drug monitoring and environmental antibiotic surveillance. Herein, nitrogen, sulfur co-doped carbon dots (N,S-CDs) were derived from L-tryptophan and thiourea for MP detection. The prepared N,S-CDs were characterized by using UV–VIS, X-ray diffraction (XRD), Fourier transform infrared (FT-IR) spectroscopy, high-resolution transmission electron microscopy (HR-TEM), and fluorescence spectroscopy. The interaction with MP led to systematic quenching of fluorescence (λ_em_ = 457 nm, λ_ex_ = 350 nm), a Stern–Volmer constant Ksv = 3.97 × 10^6^ M^−1^, a binding constant Ka = 9.07 × 10^6^ M^−1^, and a decrease in the fluorescence lifetime from 6.45 to 5.68 ns. A linear response was exhibited throughout the 0–390 nM range (R^2^ = 0.990) with a limit of detection (LOD) of 6.8 nM. The sensor demonstrated high selectivity toward MP with a 1250-fold excess of coexisting inorganic ions, biomolecules, and structurally related carbapenem antibiotics. Intracellular fluorescence imaging and concentration-dependent intracellular MP detection were also demonstrated in live PC3 cells with negligible cytotoxicity (cell viability > 80% up to 10 mg/mL), indicating its potential for clinical therapeutic drug monitoring and environmental surveillance. The probe was successfully validated for the analysis of MP in pharmaceutical formulations, human serum, urine, and environmental water samples, with recoveries (%) in the range of 98%–102%. This work presents N,S-CDs as a robust, sensitive, and biocompatible analytical tool for clinical, pharmacological, and environmental MP surveillance.

## 1 Introduction

Meropenem (MP), a broad-spectrum β-lactam carbapenem antibiotic, is a last-resort drug that can kill resistant Gram-negative bacteria (Armstrong et al., 2021; Bassetti et al., 2025; Eslami et al., 2025; Li et al., 2023; Mengesha, 2025). MP displays extensive antibacterial action and remarkable stability to most β-lactamases (Armstrong et al., 2021; Bassetti et al., 2025; Eslami et al., 2025; Li et al., 2023; Mengesha, 2025). Consequently, it finds its application in the treatment of sepsis, pneumonia, meningitis, and other deadly infections (Berry and Kuti, 2022; Li et al., 2023; Yang et al., 2025). Suboptimal doses of meropenem may result in treatment failure and risk of microbial resistance. Contrarily, higher meropenem concentrations are associated with an increased risk of dose-dependent toxicity, such as neurotoxicity (seizures) and renal effects, in critically ill patients. Still, nearly 90% of the dosed drug is emitted unchanged, which leads to high concentrations in effluents coming from hospitals (Berry and Kuti, 2022; Li et al., 2023; Ma et al., 2024; Qian et al., 2016; Zhuang et al., 2023). Unmetabolized MP and its residues are released to hospital effluents and sewage systems. It is a powerful antibiotic that ends up in sewage systems and wider environments and has the potential to possibly generate severe consequences further downstream. One of the known causes for the spread of antimicrobial resistance genes is the exposure of ambient bacteria to MP residues in water and wastewater at concentrations much lower than the lethal threshold. The constant presence of MP in the environment exerts selective pressure on microbial communities, resulting in faster emergence of carbapenem-resistant bacteria (Boutzoukas et al., 2025; Maimongkol et al., 2022). The U.S. Food and Drug Administration (FDA) determines that, based on the clinical, microbiological, and pharmacokinetic information available, an MP dosing regimen of 1 g every 8 h infused over 60 min can provide support for the following interpretive criteria for MP against *Acinetobacter*: 2 μg/mL (susceptible), 4 μg/mL (intermediate), and 8 μg/mL (resistant) (2017-N-5925-0018). As a result, MP residue levels in biological, pharmaceutical, and environmental samples should be regularly monitored for antimicrobial resistance surveillance and environmental risk assessment (Hama Aziz, 2026; Qian et al., 2016). MP has been quantified using traditional analytical methods such as high-performance liquid chromatography (HPLC) (Boateng et al., 2024; Kazanova et al., 2020; Zhang et al., 2022), electrochemical sensing (Godana et al., 2020; Jiang et al., 2018; Qin et al., 2017; Rajalakshmi and John, 2015; Zhang et al., 2026), flow cytometry (Godana et al., 2020; Ma et al., 2024; Qian et al., 2016), and liquid chromatography–mass spectrometry (LC–MS) (Federico et al., 2026). Compared with other techniques, fluorescent chemosensors have been drawing much attention (Cheng et al., 2026). They offer high sensitivity, fast reaction rates, ease of use, and real-time analysis (Gao et al., 2026; Muthusamy et al., 2025a; Muthusamy et al., 2025b; Shen et al., 2024; Tang et al., 2026b; Xiao et al., 2024). As a result, it is highly desirable to develop fluorescent probes that are selective, sensitive, and cost-effective to detect MP.

Carbon dots (CDs) are one of the most appealing materials among the many fluorescent materials due to their excellent water solubility, low toxicity, simple and low-cost preparation, tunable surface chemistry, strong fluorescence, and high sensitivity with photostability (Bai et al., 2025; Hu et al., 2019; Rajalakshmi et al., 2022; Tang et al., 2026a; Zhu et al., 2025). The abundant functional groups, such as amine, carboxyl, and hydroxyl groups, on the surface of CDs not only make them highly soluble in aqueous solutions but also enable them to be easily functionalized and contribute (Mohammed et al., 2026; Sardar et al., 2026; Wang et al., 2024; Wei et al., 2023; Xiao et al., 2024) to their extremely sensitive and tunable responses toward target analytes. Heteroatom doping has been widely used to further improve the quantum yield, surface reactivity, and analyte-binding ability of CDs (Mohammed et al., 2026; Sardar et al., 2026; Shi et al., 2024; Yang et al., 2024). This is due to the fact that the introduction of more heteroatoms leads to a larger number of surface functional groups that can participate in hydrogen bonding, electrostatic interaction, and photoinduced charge transfer with target molecules. The successful demonstration of CD-based fluorescent probes for the sensitive detection of several other antibiotics such as ciprofloxacin and tetracycline has already been demonstrated. However, to the best of our knowledge, there are no reports available thus far on the selective and sensitive detection of MP using a carbon dot probe, despite the increasing number of publications on CD-based antibiotic sensors. This is a glaring void in the literature that has not been addressed, and it is of particular concern considering the therapeutic importance of this carbapenem.

To address this gap, the present study reports for the first time the synthesis and application of nitrogen, sulfur co-doped carbon dots (N,S-CDs) as a fluorometric probe for the sensitive and selective detection of MP. L-Tryptophan was selected as the carbon and nitrogen source because its aromatic indole framework provides a preorganized conjugated structure for efficient carbonization, and its native amine and carboxyl groups contribute to surface reactivity. Thiourea was used as the nitrogen and sulfur source during the one-pot hydrothermal synthesis, enabling N,S co-doping that enriches the surface with C=O, C=N, C–N and C=S groups capable of selective non-covalent interaction with the β-lactam carbonyl, carboxylate and thioether-containing side chain of MP. This particular combination of precursors was selected to increase the interaction between the probe surface and the target analyte, differentiating them from other reported CD-based antibiotic sensors.

In this research, the synthesis, characterization, and sensing capabilities of N,S-CDs are carefully studied to evaluate whether or not they are suitable for use as a fluorometric probe for MP. The optical characteristics and structural properties of N,S-CDs were examined by using techniques such as UV-Vis absorption, fluorescence excitation/emission spectroscopy, high-resolution transmission electron microscopy (HR-TEM), powder X-ray diffraction (PXRD), and Fourier transform infrared (FT-IR) spectroscopy. The interaction of N,S-CDs with MP is investigated using fluorescence and UV–VIS titrations. Furthermore, Stern–Volmer analysis, binding constant calculation, and time-resolved fluorescence lifetime measurements are applied to further analyze the underlying quenching process. The selectivity and anti-interference ability of the probe are examined in the presence of a variety of ions, biomolecules, and structurally related antibiotics. Additionally, the biocompatibility of the probe and its applicability for intracellular MP imaging in live PC3 cells are explored. The practical use of the suggested probe was demonstrated by measuring the MP in pharmaceutical, biological, and environmental water samples and comparing it with a reference HPLC method. Overall, the aim is to develop N,S-CDs as a sensitive, selective, and biocompatible platform for MP monitoring in clinical, pharmacological, and environmental applications. The results of this work, taken together, demonstrate N,S-CDs as a novel fluorescent nanoprobe with good sensitivity, selectivity, and biocompatibility.

## 2 Experimental section

### 2.1 Synthesis of N,S-CDs

N,S-doped carbon dots (N,S-CDs) were synthesized via a straightforward one-step hydrothermal method utilizing L-tryptophan as a source of carbon and nitrogen and thiourea as a source of nitrogen and sulfur. In summary, L-tryptophan (0.05 g, 0.245 mmol, MW = 204.23 g/mol) and thiourea (0.02 g, 0.263 mmol, MW = 76.12 g/mol) were solubilized in 50 mL of deionized (DI) water with magnetic stirring until a homogeneous clear solution was achieved. The acquired solution was placed in a Teflon-lined stainless-steel autoclave (100 mL) and subjected to hydrothermal treatment at 180 °C for 6 h in a muffle oven. Subsequent to the reaction, the autoclave was allowed to cool to ambient temperature naturally. The resultant brown-yellow solution was collected and subjected to centrifugation many times at a relative centrifugal force (rcf) of 12000 to remove larger particles. The supernatant was meticulously collected and filtered through a 0.2 µm syringe filter. Subsequent purification was conducted using dialysis for 48 h against deionized water, with a 3.5 kDa molecular weight cutoff dialysis membrane, with frequent water exchanges to remove unreacted precursors and small molecules. The final purified NS-CD solution was stored at 4 °C and employed for subsequent characterization and sensing assessments.

### 2.2 Spectroscopy measurements

The UV-Vis absorption spectra of N,S-CDs were measured in DI water at a concentration of 0.4 mg/mL. Fluorescence emission spectra were recorded in DI water at a concentration of 0.2 mg/mL. For the MP sensing experiments, the fixed concentration of N,S-CDs (0.2 mg/mL) was employed as the probe. MP and all analytes were freshly dissolved in DI water before use. During selectivity research, all possible interferents were separately introduced to the solution of N,S-CDs, and the corresponding fluorescence emission response was recorded. The interference (anti-interference) investigation was carried out by adding each interferent to a combination of N,S-CDs and MP at a predetermined concentration and monitoring the change in fluorescence emission.

### 2.3 Sample preparation for real sample analysis

The efficacy of the developed N,S-CD fluorescent probe for detecting MP was evaluated in real biological and pharmaceutical samples, including MP injection (pharmaceutical formulation), human blood serum, and human urine specimens. The commercial MP injection (Kefen®, 0.5 g/vial) was reconstituted with DI as directed and appropriately diluted prior to analysis. Human blood serum samples were obtained from healthy individuals, centrifuged at 3000 rcf for 10 min to remove particulates, and diluted 20-fold with deionized water before the sensing experiment. Urine samples from healthy subjects were collected, filtered via a 0.2 μm membrane, and diluted 20-fold with deionized water before analysis. The quantified amounts of MP were incorporated into each genuine sample matrix at three concentration levels, and the recovery was assessed using the following formula. All experiments were performed in five replicates (n = 5), and the mean values were reported. The obtained results were further validated against the conventional HPLC method:

$$\text{Recovery }\left(\%\right)=\left(\text{Detected concentration }/\text{ Spiked concentration}\right)\times 100.$$

The concentration of MP determined by the proposed N,S-CD fluorimetric method was cross-validated using a reference HPLC method. The analysis was performed using an Agilent Zorbax Eclipse Plus C18 column (150 × 4.6 mm, 5 μm) maintained at 30 °C. The mobile phase consisted of acetonitrile and 10 mM phosphate buffer (pH 7.0) (50:50, v/v), delivered isocratically at a flow rate of 1.0 mL/min. Detection was carried out at 298 nm, with an injection volume of 10 µL. Under these conditions, MP eluted at a retention time of approximately 3.85 min, with a total run time of 10 min. Sample pretreatment for HPLC analysis followed the same protocol.

Detailed materials and methods, instrumentation, cell culture, cytotoxicity, and live cell imaging details were provided in the supporting information.

## 3 Results and discussion

### 3.1 Optimization of synthesis of N,S-CDs

N,S-CDs were synthesized by a simple hydrothermal method in a one-pot manner using L-tryptophan and thiourea as precursors. The aromatic base of tryptophan, with its indole group, offers amine and carboxyl groups along with a natural carbon and nitrogen skeleton. Thiourea, conversely, offers a complementary source of nitrogen and sulfur, thereby allowing co-doping during the carbonization process. A detailed description of the synthetic technique is given in Section 2.1. The primary reaction parameters, such as the excitation wavelength, hydrothermal temperature, and reaction time, were systematically varied and evaluated according to the emission response obtained. This was done to obtain N,S-CDs with the best feasible fluorescence performance. The excitation–emission spectrum of the N,S-CDs in their prepared state is shown in Supplementary Figure S1A. It was observed that there are two excitation maxima at about 270 and 355 nm corresponding to π–π* and n–π* transitions, respectively. On the contrary, the emission spectrum consisted of a single band, well-resolved, at 457 nm. The optimal excitation condition was obtained by scanning the probe over an excitation range of 330–360 nm (Supplementary Figure S1B). The emission intensity increased gradually while exciting from 330 to 350 nm and then declined with a considerable bathochromic shift. This pattern correlates with the heterogeneous distribution of surface emissive states typical of doped carbon dots. Hence, 350 nm was chosen as the working excitation wavelength.

All other parameters were then preserved at their initial values while the temperature of the hydrothermal process was altered from 160 °C to 190 °C (Supplementary Figure S1C). The fluorescence intensity increased constantly with temperature up to 180 °C, indicating better development of the linked carbon core and more successful carbonization. However, the intensity dropped when the temperature was elevated to 190 °C. This was probably caused by over-carbonization and partial loss of surface-bound functional groups. Likewise, the reaction time was optimized at a constant temperature of 180 °C for a duration of 5–8 h (Supplementary Figure S1D). After 6 h, the signal began to sink down, indicating that protracted heating promotes aggregation and self-quenching instead of boosting the optical output. In order to directly see the effect of intensive heat treatment during the synthesis, transmission electron microscopy (TEM) images were acquired of N,S-CDs (Supplementary Figure S2). As the temperature reached 180 °C, the particles remained tiny, spherical, and well-dispersed (Supplementary Figure S2A). In contrast, samples that were subjected to 190 °C demonstrated a considerable clustering of particles into larger and more irregular aggregates (Supplementary Figure S2B). Aggregation is consistent with enhanced interparticle fusion and partial degradation of surface-passivating functional groups under prolonged thermal exposure. This enhances non-radiative energy dissipation routes (aggregation-caused quenching), which accounts for the reduction in fluorescence intensity observed beyond the ideal reaction conditions (Supplementary Figures S1C, S1D). All these results demonstrated that the optimum conditions for the synthesis of N,S-CDs were an excitation wavelength of 350 nm, a hydrothermal temperature of 180 °C, and a reaction time of 6 h. These settings were used throughout the rest of the investigation.

### 3.2 Characterizations of prepared N,S-CDs

The optimal synthesis conditions (Section 3.1; 180 °C, 6 h) were chosen to fabricate the N,S-CDs, which were used in all subsequent structural and optical characterization experiments. The UV–VIS absorption spectra of N,S-CDs presented three different absorption bands displayed in Supplementary Figure S3. The strongest of these bands was at approximately 230 nm, with the other two at approximately 270 and 350 nm. The band with high energy at 230 nm can be assigned to the π–π* transition of the aromatic C=C framework, which originates from the indole moiety of tryptophan, consistent with the heteroatom-enriched surface chemistry of the N,S co-doped carbon dots. Furthermore, the bands at 270 and 350 nm are due to the n–π* transitions of the C=O, C=N, and C=S functional groups, which may have been introduced by thiourea during carbonization. The excitation and emission spectra of N,S-CDs are shown in Supplementary Figure S1A. Two excitation maxima centered at 270 and 355 nm were observed, confirming that these electronic transitions are the cause of the probe’s fluorescence activity. It was discovered that these maxima closely matched the measured absorption properties (Supplementary Figure S3). The excitation at 350 nm produced a single, symmetric emission band centered at 457 nm (Tang et al., 2026a).

HR-TEM (Figure 1) was carried out to further examine the shape and size distribution of N,S-CDs. The TEM micrographs (Figures 1A–C) show well-dispersed, spherical nanoparticles without significant aggregation, confirming the successful fabrication of unique carbon dot nanostructures under the optimal hydrothermal conditions. The corresponding particle size histogram (Figure 1D) had been constructed from individual particles and indicated high levels of size homogeneity with a size distribution between 2.2 and 8.2 nm, an average particle diameter of 4.56 ± 1.11 nm, and the largest population centered at 4.8 nm. The crystalline nature of the as-synthesized N,S-CDs was investigated by PXRD. The diffraction pattern shows a single broad hump centered at 2θ = 26.2°, as shown in Figure 2A. This hump is characteristic of amorphous, disordered graphitic carbon, which results from the (002) interlayer spacing of stacked sp2 carbon sheets. The absence of sharp and well-defined diffraction peaks suggests that the N,S-CDs have an amorphous carbon framework rather than a highly organized crystalline structure. This is consistent with the typical structural features that have been described for carbon dots synthesized by hydrothermal methods (Arul et al., 2023a; 2023b).

**FIGURE 1:**
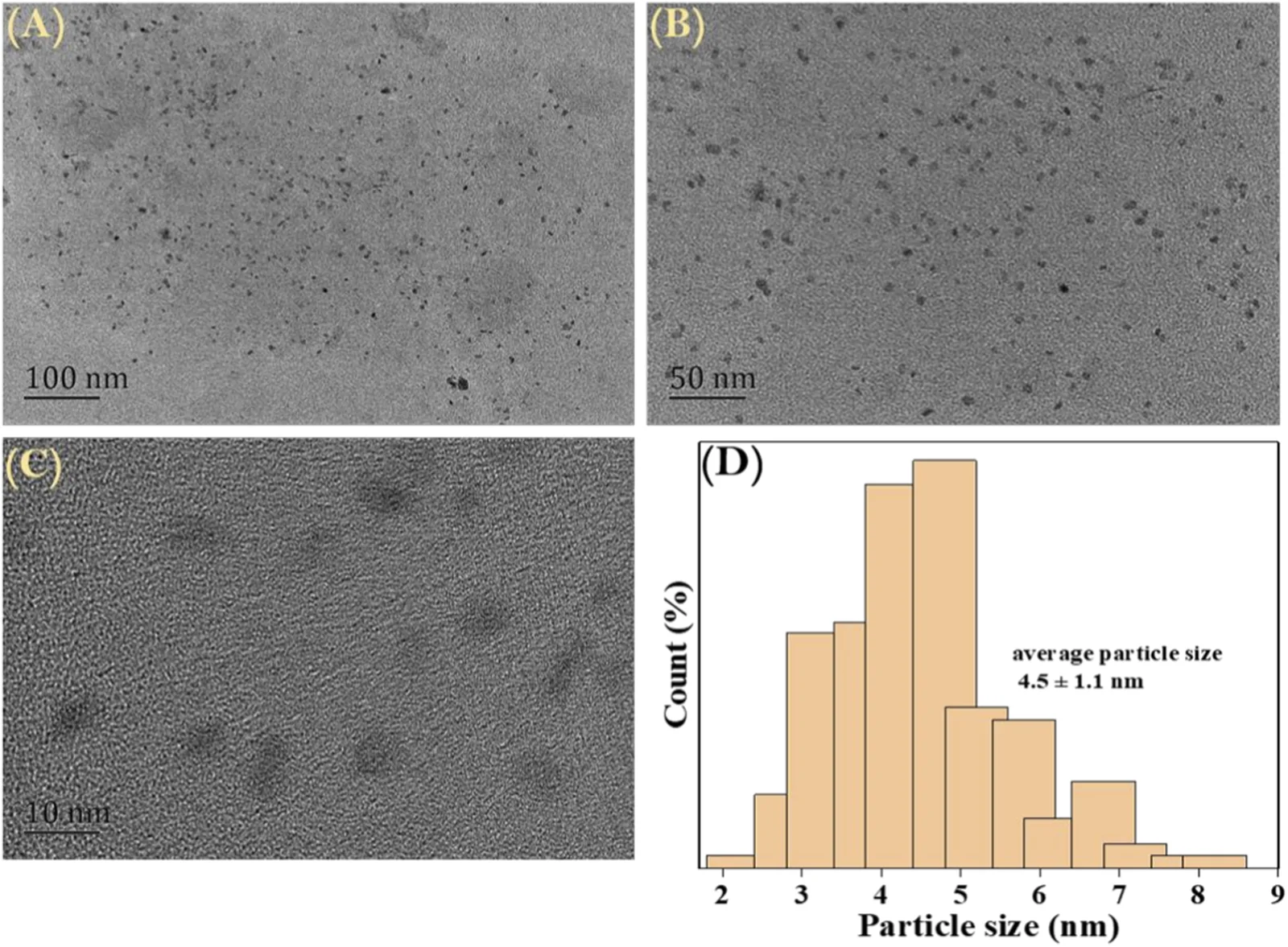
**(A–C)** High-resolution TEM images and **(D)** the particle size distribution obtained for N,S-CDs.

**FIGURE 2:**
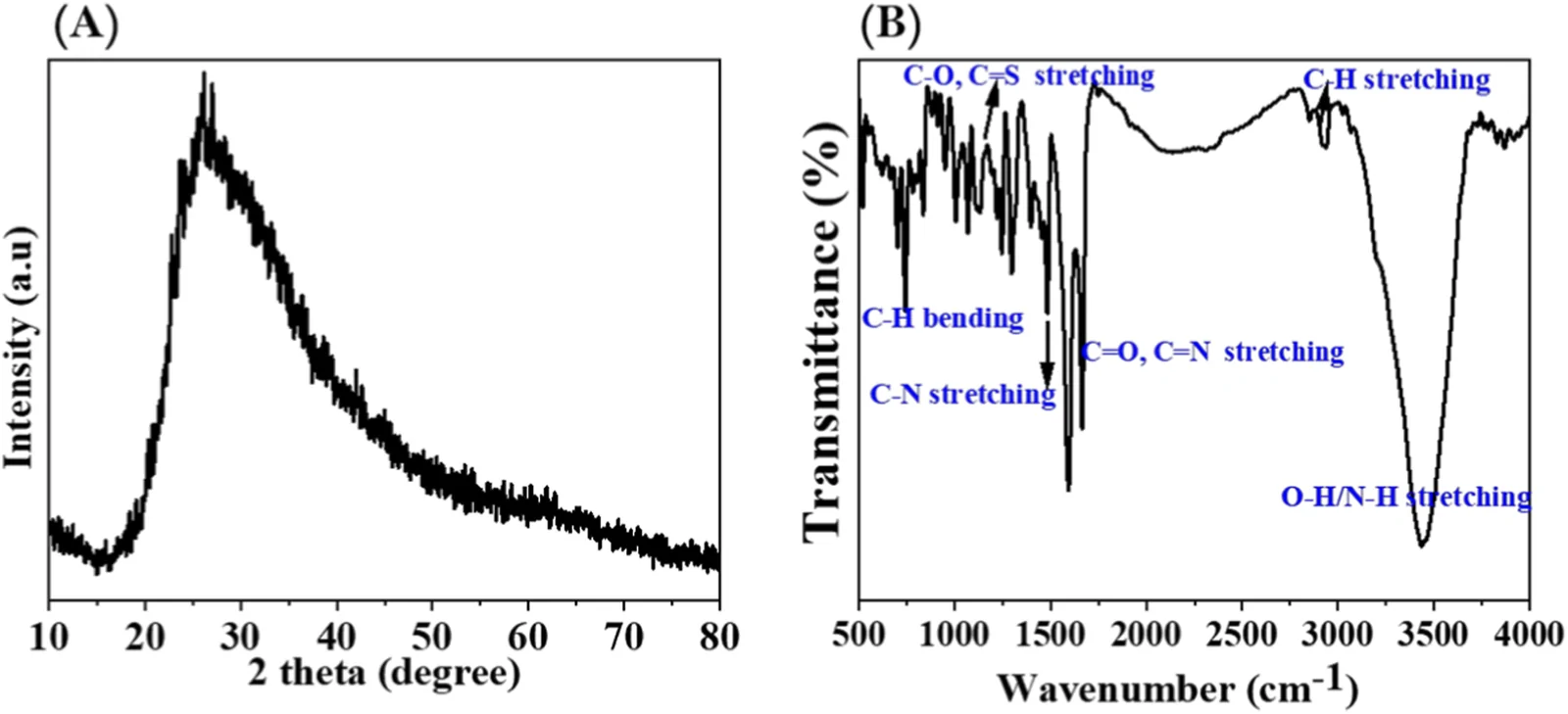
Characterizations of N,S-CDs through the **(A)** XRD spectrum and **(B)** FT-IR spectrum.

The surface functional groups that were present on the N,S-CDs were further elucidated by the use of FT-IR spectroscopy (Figure 2B). The broad and strong band centered at approximately 3,400 cm^−1^ was attributed to the presence of hydroxyl and amine/amide functional moieties on the surface of the carbon dot. This band was linked to the overlapping stretching vibrations of O–H and N–H groups. Bands in the region of 2,800–3,000 cm^−1^ correspond to stretching vibrations between carbon and hydrogen. The lower intensity bands, located at 1,380–1,450 cm^−1^, were assigned to C–N stretching, while the C–H bending vibration was observed at a lower wavenumber. The bands in the region 1,600–1,700 cm^−1^ were linked to overlapping C=O and C=N stretching vibrations. The bands in the fingerprint region of 1,000–1,300 cm^−1^ confirmed the successful inclusion of sulfur into the carbon dot framework. These bands have been attributed to the overlapping stretching vibrations of carbon–oxygen and carbon–sulfur (Bai et al., 2025; Karim and Aziz, 2025; Mustafa and Aziz, 2023; Zhu et al., 2026). The results of the UV-Vis absorption, fluorescence excitation/emission, HR-TEM, PXRD, and FT-IR investigations collectively revealed that the synthesis of N,S-CDs has been successfully achieved. These N,S-CDs have an amorphous, graphitic carbon core adorned with a variety of surface functional groups that include oxygen, nitrogen, and sulfur (–OH, –NH, C=O, C=N, C–N, and C=S). It is expected that these surface moieties will play an important role in the reactivity of N,S-CDs with MP via hydrogen bonding and other non-covalent interactions. This will confirm their suitability for further application as a fluorometric probe for the sensitive and selective detection of MP.

### 3.3 Detection of MP using N,S-CDs

The initial demonstration of MP detection using N,S-CDs was achieved via UV-Vis spectroscopy (Figure 3A). The absorption bands at 230, 270, and 350 nm wavelengths were observed for the N,S-CDs that are conventional for heteroatom-doped CDs. By adding 0.2 µM of MP, the bands at 230 and 350 nm were decreased. Notably, the peak at 270 nm was increased with a redshift. The absorbance increased gradually with the progressive addition of MP (each addition was 0.2 µM); the absorbance at 230 and 350 nm decreased, and a new peak appeared and increased at 298 nm, which is caused by the intrinsic absorption of MP. MP, which has a β-lactam ring fused with a pyrrolidine ring and a side chain containing a thioether, is known to characteristically absorb at 298 nm. The observed absorbance at 298 nm thus represents the added absorbance of MP, which increases with increasing concentration. Conversely, the decrease at 350 nm, which is specific to the C=O, C=N, and C=S surface functional groups of N,S-CDs and is not absorbed by MP, suggests a real ground-state interaction between N,S-CDs and MP. A clearly defined isosbestic point at 280 nm was observed during titration, which confirmed direct, specific ground-state complexation and not a random or non-specific contact. This was shown by the obvious balance between the two absorbing species, namely, free N,S-CDs and the N,S-CD–MP complex.

**FIGURE 3:**
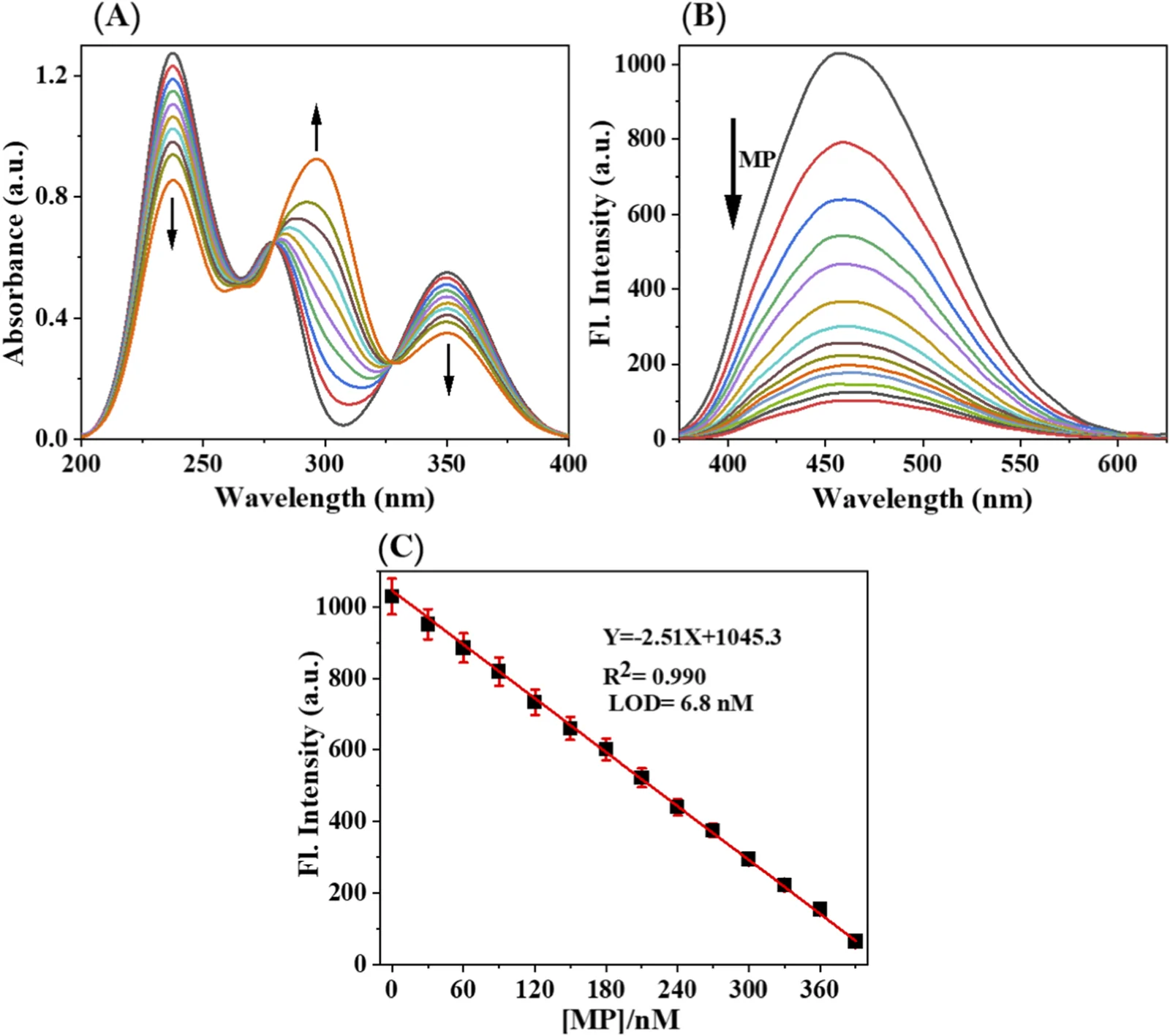
**(A)** UV–VIS spectra obtained for N,S-CDs and their reaction with each gradual addition of 0.2 µM of MP. **(B)** Fluorescence emission spectra obtained for N,S-CDs in the presence of various concentrations of MP (each 30 nM). **(C)** Linearity plot of fluorescence intensity against MP concentration (n = 3).

To further evaluate the sensitivity of the probe toward MP detection, a fluorescence spectroscopy titration was performed (Figure 3B). It was observed that N,S-CDs had an emission maximum at 457 nm upon excitation at 350 nm. For each addition of 30 nM of MP, a progressive and systematic drop in fluorescence intensity was observed. The quenching reaction can be attributed to the combination of ground-state complex formation between N,S-CDs and MP, which is in conformity with the isosbestic point found in the UV–VIS titration. Moreover, this fluorescence quenching mechanism is attributed to photoinduced charge or energy transfer from the electron-rich surface functional groups (–OH, –NH, C=O, C=N, C–N, and C=S) of N,S-CDs to the electron-deficient β-lactam carbonyl and thioether groups of MP. Moreover, the partial spectral overlap between the excitation band of N,S-CDs and the absorption band of MP at 298 nm implies that the inner-filter effect is not the only reason responsible for the phenomenon. A detailed investigation of the mechanism is presented in Section 3.4, with the quenching constant and the binding constant of the quenching process. Figure 3C illustrates the correlation between fluorescence intensity and MP concentration. The linear regression equation Y = −2.51X + 1045.3, having a correlation coefficient (R^2^) of 0.990, was utilized to ascertain the reduction of fluorescence intensity within the range of 0–390 nM. The LOD was determined using the formula LOD = 3σ/S, where σ represents the standard deviation of the blank signal and S is the slope of the calibration curve. The calculated value was 6.8 nM, which validates the superior sensitivity of the N,S-CD probe to MP. The obtained LOD was compared with several carbon dot-based spectrofluorimetric methods published in the literature for the determination of MP (Table 1). This comparison demonstrates that the new N,S-CD probe exhibits comparable sensitivity for MP detection to the earlier reports (Elzanfaly et al., 2017; Fayed et al., 2019; 2021).

**TABLE 1:** Comparison of the proposed N,S-CD fluorimetric sensor with previously reported spectrofluorimetric methods for the determination of MP.

S.No	Sample	Linear range (nM)	LOD (nM)	Reference
1	gNPs-enhanced fluorescence (MP–Eu^3+^ chelate)	10.4–36.5 nM	2.2 nM	Fayed et al. (2021)
2	NBD-Cl derivatization fluorometric method	26.1–912.7 nM	5.6 nM	Fayed et al. (2019)
3	Tb^3+^ chelation – zero-order synchronous spectrofluorimetry	521.6–26078.3 nM	NA	Elzanfaly et al. (2017)
4	**N,S-CDs (this work)**	**0–390 nM**	**6.8 nM**	**This work**

Bold values indicate the results obtained in this work. NA, not available.

### 3.4 Quenching mechanism, quenching, and binding constants

Stern–Volmer (SV) analysis was used to further study the quenching behavior of N,S-CDs with MP. The purpose of this study was to elucidate the quenching and binding parameters associated with the quenching process (Supplementary Figure S4A). The SV plot was constructed by applying the formula F_0_/F = 1 + Ksv[Q] where F_0_ and F are the fluorescence intensities of N,S-CDs without and with MP, respectively, and [Q] is the concentration of MP. The SV plot exhibited a considerable upward divergence from linearity at higher concentrations of MP. This non-linear pattern suggests the simultaneous operation of both dynamic and static quenching mechanisms, as opposed to the existence of a single quenching pathway. The Stern–Volmer quenching constant (Ksv) was found to be 3.97 × 10^6^ M^−1^ from the initial linear area at low concentrations of MP. This result indicates the great quenching efficiency of MP toward N,S-CDs. The binding constant (Ka) for the ground-state interaction between N,S-CDs and MP was carried out using the modified double-logarithmic Stern–Volmer equation. The equation is stated as log[(F_0_ − F)/F] = logKa + n log[Q], where n is the number of binding sites. The linear plot of Supplementary Figure S4B indicated the value of Ka to be 9.07 × 10^6^ M^−1^ and the binding stoichiometry (*n*) obtained to be 1.81. The relatively high Ka value shows that the ground-state interaction between N,S-CDs and MP is quite stable. Conversely, an *n* value greater than unity suggests that more than one binding/interaction site on the heterogeneous N,S-CD surface is probably involved in accommodating each MP molecule. This is consistent with the various surface functional groups (–OH, –NH, C=O, C=N, and C=S) found by FT-IR analysis.

The static and dynamic quenching features of the observed fluorescence quenching were clearly distinguished by performing time-resolved fluorescence lifetime measurements for N,S-CDs in the absence and presence of MP (Figure 4). Interaction with MP decreased the average fluorescence lifetime of N,S-CDs from 6.45 to 5.68 ns. In static quenching, complex formation in the ground state, which is non-fluorescent, does not impact the intrinsic excited-state lifetime of the remaining free fluorophore. Conversely, dynamic (collisional) quenching leads to a direct decrease of the reported lifetime via an extra non-radiative decay channel. Furthermore, the decrease in lifetime indicates the contribution of a dynamic quenching component. This is in addition to the static component that was already suggested by the isosbestic point in the UV–VIS titration (Section 3.3) and the upward curvature of the SV plot.

**FIGURE 4:**
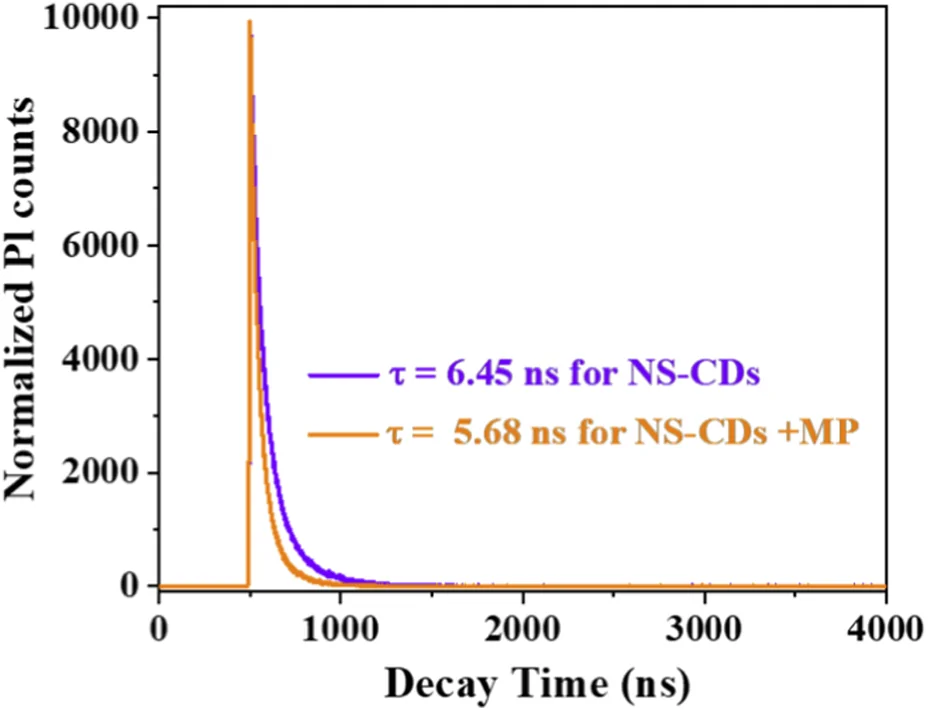
Fluorescence lifetime decay curves obtained for N,S-CDs (violet line) and N,S-CDs with MP (orange line).

Overall, the isosbestic point in the UV–VIS spectra, the upward-curving SV plot along with the binding constant analysis, and the decrease in fluorescence lifetime demonstrate that MP suppresses the fluorescence of N,S-CDs through a combination of static and dynamic processes. There is a static contribution due to the creation of a ground-state complex. The synthesis of this complex is most likely mediated by hydrogen bonding and electrostatic interactions between the surface –OH, –NH, C=O, C=N, and C=S functional groups of N,S-CDs and the β-lactam carbonyl, carboxylate, and thioether moieties of MP. The dynamic part originates from the collisional, photoinduced charge, or energy transfer between MP and excited-state N,S-CDs. It is also probable that there is a partial inner-filter effect due to the spectral overlap between the excitation band of N,S-CDs and the absorption band of MP at 298 nm, which may also play a marginal role in the overall quenching efficiency. The inner-filter effect was evaluated qualitatively and was not quantitatively corrected.

### 3.5 Selectivity and interference ability of N,S-CDs

The selectivity of the fluorometric probe to respond to the target analyte in the presence of structurally similar compounds and/or common coexisting species in real sample matrices is an important consideration for practical applications of the probe. To test the selectivity and anti-interference capabilities of N,S-CDs toward MP, three types of possible interferers were carefully explored. The categories comprised typical inorganic cations and anions; physiologically important amino acids and sugars, which are present in common human body fluids; and structurally similar antibiotic medicines, which included numerous different carbapenem antibiotics (Supplementary Figure S5). The selectivity response was tested by incubating N,S-CDs with 0.5 mM of each interferent (including MP itself) independently and monitoring the fluorescence intensity. The anti-interference response was investigated by adding 0.5 mM of each interferent to a fixed mixture of N,S-CDs and MP (0.4 µM) and recording the change in fluorescence intensity variations. The fluorescence intensity of N,S-CDs did not vary significantly with the addition of the cations Cu^2+^, Fe^3+^, Pb^2+^, Hg^2+^, Cd^2+^, Ca^2+^, Mg^2+^, and Zn^2+^ and the anions Cl^−^, Br^−^, NO_3_^−^, NO_2_^−^, SO_4_^2−^, CH_3_COO^−^, and SCN^−^ (selectivity bars) as shown in Supplementary Figure S5A. Conversely, MP alone caused a significant and reproducible drop in fluorescence intensity. Subsequently, the fluorescence intensity was measured when each ion was added together with MP (interference bars) and sustained at a level closely comparable to that of N,S-CDs alone treated with MP. These results demonstrate that none of the examined ions interfered with or restored the fluorescence response provided by MP.

A similar pattern was observed for the panel of amino acids (Gly, Ala, Cys, Lys, His, Arg, Glu, and Asp) and saccharides (glucose, fructose, sucrose, and lactose) (Supplementary Figure S5B), as well as for the panel of antibiotic drugs that are structurally related and clinically co-administered. These antibiotic drugs include levofloxacin, tetracycline, doxycycline, doripenem, ampicillin, imipenem, ertapenem, amoxicillin, ciprofloxacin, chloramphenicol, gentamicin, and vancomycin (shown in Supplementary Figure S5C). One thing that is particularly noteworthy is that doripenem, imipenem, and ertapenem, which are all members of the same carbapenem subclass as MP and have the same characteristic β-lactam core along with a thioether-containing side chain, did not produce any appreciable fluorescence quenching of N,S-CDs when they were tested individually. Furthermore, when they were present in combination with MP, they did not significantly alter the MP-induced quenching response. According to this discovery, the sensing response that is observed is not solely governed by the general carbapenem scaffold; rather, it is dependent on particular structural features that are exclusive to MP. This finding is in line with the selective non-covalent interactions, hydrogen bonding, and electrostatic association that are discussed in Section 3.4 between the surface functional groups of N,S-CDs and MP. After extensive studies, it was found that N,S-CDs were highly selective for MP even in the presence of a range of cations, anions, biomolecules, and structurally related antibiotics including the closely related carbapenems. This is true even when the interferent-to-analyte concentration ratio is significant, such as 0.5 mM interferent vs 0.4 μM MP, which represents a 1250-fold excess. These results have validated the excellent anti-interference capabilities of N,S-CDs and proved that these CDs are suited for the accurate detection of MP in complex real sample matrices.

### 3.6 Effect of pH and reaction time on the detection of MP using N,S-CDs

Sensing performance of a fluorometric probe is often influenced by environmental factors such as solution pH and the reaction time required to reach a stable signal, so the influence of these parameters was systematically studied for N,S-CDs with and without MP to establish the optimal and reproducible sensing conditions. The effect of pH on fluorescence strength of N,S-CDs was studied in the absence and presence of 0.4 µM MP. This study was performed over a pH range of 3–10 as shown in Figure 5A. The fluorescence intensity of N,S-CDs was extremely pH-dependent in the absence of MP. It climbed progressively from pH 3 to a maximum at pH 7 and then fell gradually until pH 10. The reason for this trend is the protonation/deprotonation equilibria of the surface functional groups (–OH, –NH, and –COOH) of N,S-CDs in acidic and basic media, which change the surface charge distribution. Generally, the carbon dots are most stable and most emissive in neutral conditions. A similar pH-dependent trend was seen for N,S-CDs in the presence of MP. The fluorescence intensity was continuously lower than that of N,S-CDs alone and considerably lower over the whole pH range. This result further supports the presence of MP-induced quenching independent of the pH environment. It should be noted that the highest difference in the fluorescence intensities of N,S-CDs and N,S-CDs with MP was seen at pH 7. This shows that the sensing response of N,S-CDs for MP is most easily observed under physiologically relevant and neutral conditions. Thus, pH 7 was selected as the optimum working pH for all subsequent sensing analysis. This is especially useful in view of the fact that the probe has to be utilized in physiological matrices such as serum and urine samples.

**FIGURE 5:**
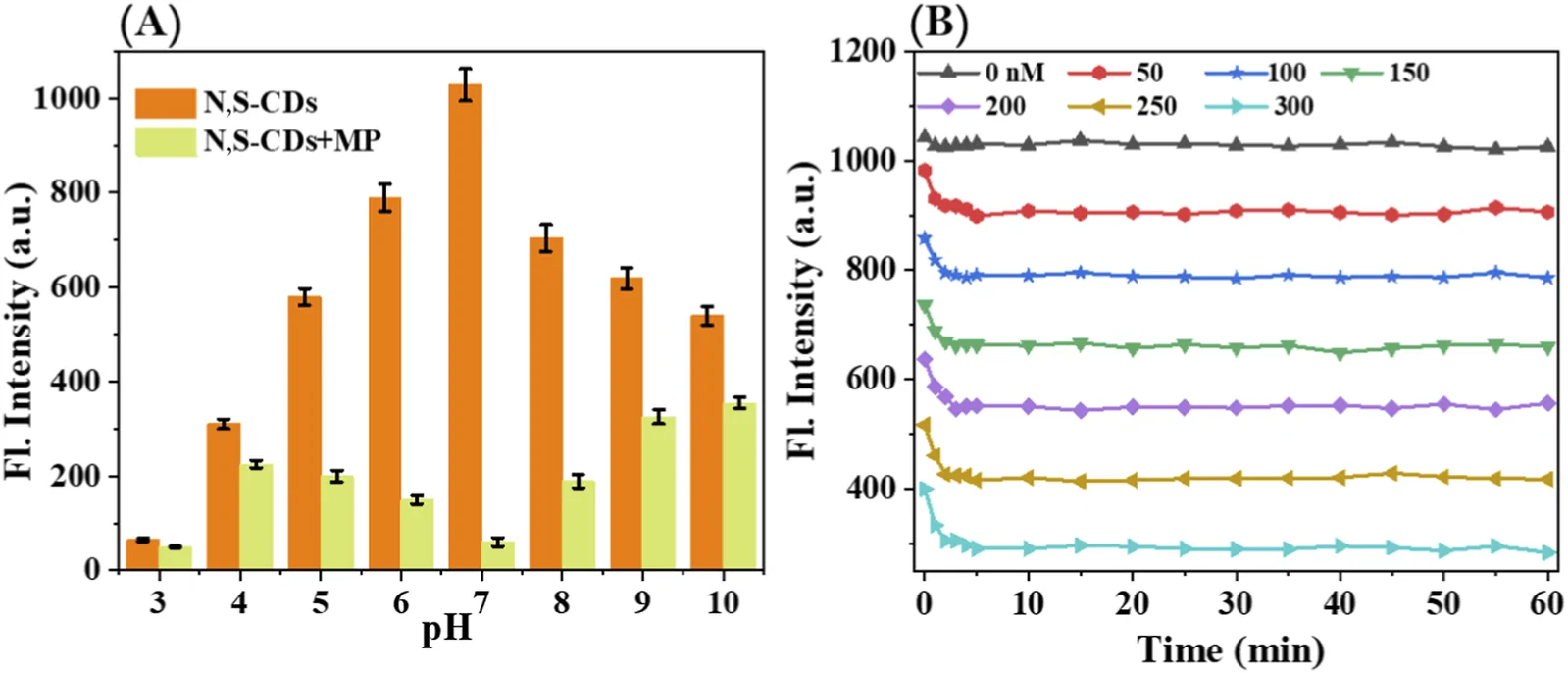
Effect of pH on N,S-CDs **(A)** in the absence and presence of 0.4 µM of MP and **(B)** plot of fluorescence emission intensity versus reaction time obtained for N,S-CDs in the presence of different concentrations of MP (n = 3).

The necessary response time for N,S-CDs to achieve a stable fluorescence signal upon interaction with MP was assessed by monitoring the fluorescence intensity for a period of 60 min at varying concentrations of MP (0, 50, 100, 150, 200, 250, and 300 nM) (Figure 5B). Within the first several minutes of MP addition, the fluorescence intensity of N,S-CDs decreased substantially for all the doses examined. Then, the intensity of the fluorescence approached a steady plateau, which stayed fixed for a period of up to 60 min with no noticeable further change. The rapid attainment of equilibrium implies fast complexation kinetics between N,S-CDs and MP, consistent with the static, ground-state interaction described in Section 3.4. From these results, it was decided that a 5 min reaction time was sufficient to allow complete and reproducible quenching of fluorescence. This incubation time of 5 min was employed for all subsequent MP evaluations with N,S-CDs.

The influence of temperature on the fluorescence response of N,S-CDs to MP was also studied in the range of 15 °C–40 °C (Supplementary Figure S6). The fluorescence intensity monotonically decreased with increasing temperature, which is in line with the characteristic thermally induced non-radiative decay processes of carbon dot fluorophores. The MP-induced quenching response was found to be strong under subambient and near-physiological conditions, as it remained distinct over the range studied. Therefore, room temperature (25 °C) was selected as the working temperature for all subsequent sensing experiments, as it is convenient and suitable for detection in real and biological samples.

### 3.7 Cytotoxicity and bioimaging applications of N,S-CDs

The biocompatibility of N,S-CDs was evaluated before investigating their potential as intracellular MP sensors, in view of the possible application of N,S-CDs in biological systems. The cytotoxicity of N,S-CDs against PC3 cells was investigated by the conventional MTT analysis. For this, different amounts (0, 0.2, 0.5, 1, 3, 5, and 10 mg) of N,S-CD powder were each dissolved in 1 mL of deionized water to give concentrations of 0–10 mg/mL (n = 5), and the cells were treated with these differently concentrated N,S-CDs. The cell survival ratio achieved from this experiment was also measured (Supplementary Figure S7). Cell viability was kept above 95% at N,S-CD concentrations up to 0.5 mg/mL, indicating that there was no significant cytotoxic effect at lower to moderate doses at these concentrations. With the increase in concentration of N,S-CDs, a progressive and dose-dependent decrease in cell viability was observed. At the highest tested dose (10 mg/mL), the survival ratio was reduced to approximately 81%. Interestingly, even at this highest concentration, cell viability was still above 80%, which is typically considered to be an acceptable threshold for biocompatibility in cell-based experiments. This indicates that N,S-CDs have low cytotoxicity in the whole studied concentration range; hence, they are appropriate for further bioimaging applications.

To further examine the potential of N,S-CDs for intracellular fluorescence imaging and MP sensing in living cells, confocal laser scanning microscopy was employed on PC3 cells (Figure 6). This analysis was carried out to expand on the previously indicated good biocompatibility profile. PC3 cells treated with N,S-CDs alone (10 and 30 μL of 0.2 mg/mL) exhibited intense and distinct blue fluorescence, mostly localized in the cytoplasmic portion of the cells (Figures 6A,D). The N,S-CD-tagged cells subsequently treated with MP showed a clear decrease in the intracellular blue fluorescence intensity. The decline was observed to be concentration-dependent. The fluorescence signal was obviously lower than that of the control containing only N,S-CDs after treatment with 1 µM of MP (Figures 6B,E). In addition, treatment with a higher MP concentration of 3 µM resulted in an even greater reduction in fluorescence intensity with cells appearing much darker (as seen in Figures 6C,F). This progressive, concentration-dependent quenching of intracellular fluorescence is very similar to the quenching behavior observed in solution-phase fluorescence titration experiments (Section 3.3), demonstrating that the MP sensing ability of the N,S-CDs is retained within a living cellular environment. The above results collectively demonstrate that N,S-CDs exhibit low cytotoxicity for PC3 cells and intracellular fluorescence localization and may be used as effective intracellular fluorescent probes for visual and concentration-dependent detection of MP. This suggests that N,S-CDs have potential for bioimaging-based tracking of MP in live systems.

**FIGURE 6:**
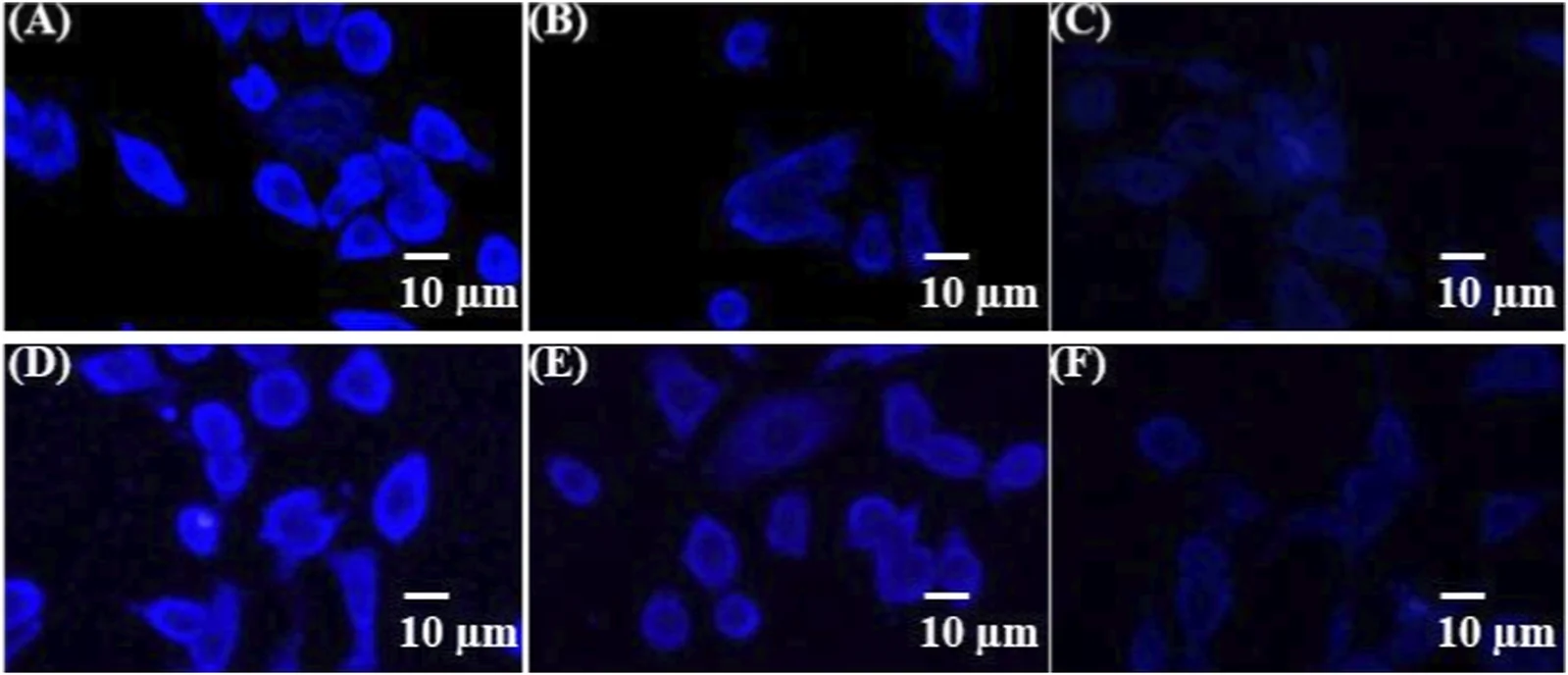
Fluorescent confocal microscopic images of PC3 cells: **(A,D)** PC3 cells treated with 10 and 30 µL of N,S-CDs (0.2 mg/mL) only. **(B,E)** N,S-CD-stained cells treated with 1 µM of MP. **(C,F)** N,-CD-stained cells treated with 3 µM of MP.

### 3.8 Real sample analysis

To evaluate the practical usability of the proposed N,S-CD fluorimetric probe, the quantitative analysis of MP was conducted in five practical samples including MP injection (Kefen®), human serum, human urine, tap water, and pond water. The samples were infused with specified amounts of MP at three concentration levels (20, 50, and 100 nM), all of which were within the probe’s linear range. The recoveries were ascertained by contrasting the measured concentrations with the spiked levels. The collected data were subsequently validated against a reference HPLC technique. As presented in Table 2, the developed N,S-CD fluorimetric probe afforded good and reproducible recoveries in all five sample matrices. In addition, the probe was able to produce these recoveries at three spiking concentration levels in the defined linear range. The fluorimetric readings throughout the full process showed good agreement with the results of the reference HPLC method. All matrices had recovery values in the analytically acceptable range of 98%–102%. The estimated Student’s t-test and F-test findings were still below their respective tabulated critical values at the 95% confidence level. This shows that there is no substantial difference between the suggested approaches and the reference methods in accuracy and precision. Together, our results confirm the practical utility and matrix tolerance of the N,S-CD probe for reliable MP determination in a variety of practical sample matrices, including those relevant to the pharmaceutical, biological, and environmental applications.

**TABLE 2:** Real sample analysis of MP using the proposed N,S-CD fluorimetric method compared with HPLC (n = 5).

Samples	MP spiked (nM)	MP measured (nM)		Recovery (%)
		Fluorimetry method	HPLC method	
Kefen injection	20	19.77	20.03	98.85
​	50	50.51	49.42	101.02
​	100	99.55	98.27	99.55
Human serum	20	20.05	19.80	100.25
​	50	50.55	49.95	101.10
​	100	99.73	99.90	99.73
Human urine	20	19.72	19.96	98.60
​	50	49.25	49.19	98.50
​	100	100.15	100.24	100.15
Tap water	20	20.16	19.66	100.80
​	50	49.90	50.28	99.80
​	100	99.66	97.97	99.66
Pond water	20	20.24	19.96	101.20
​	50	49.15	49.68	98.30
​	100	101.13	98.37	101.13

## 4 Conclusion

In summary, nitrogen, sulfur co-doped carbon dots (N,S-CDs) were effectively synthesized as a novel fluorimetric nanoprobe for the sensitive and selective sensing of meropenem (MP). The N,S-CDs were synthesized by a one-pot hydrothermal procedure using L-tryptophan and thiourea. By strategically selecting precursors through the combination of the aromatic indole backbone of L-tryptophan and the dual nitrogen-sulfur donation of thiourea, the well-defined amorphous graphitic N,S-CDs with a diameter of approximately 4.56 ± 1.11 nm were obtained. These N,S-CDs have a surface functionality that was complementary to the structural properties of MP. The fluorescence quenching of N,S-CDs by MP was confirmed to involve combined static and dynamic quenching mechanisms by Stern–Volmer analysis (Ksv = 3.97 × 10^6^ M^−1^), binding constant determination (Ka = 9.07 × 10^6^ M^−1^, n = 1.81), UV-Vis isosbestic point, and fluorescence lifetime reduction (6.45 → 5.68 ns). The proposed probe shows a linear sensing range of 0–390 nM with a detection limit (LOD) of 6.8 nM. High selectivity was obtained even in the presence of a 1250-fold excess of coexisting interferents. Validated recoveries of 98%–102% were obtained from pharmaceutical, biological, and environmental water matrices; the values were validated using a high-performance liquid chromatography (HPLC) method. The additional evidence of insignificant cytotoxicity and intracellular fluorescence-based MP detection in live PC3 cells has confirmed N,S-CDs as a trustworthy, biocompatible analytical platform with broad applications including clinical therapeutic drug monitoring, pharmaceutical quality control, and environmental antibiotic surveillance. Future work will cover the structural validation of the N,S-CD–MP interaction and testing on complex wastewater matrices and other cell models, which will enable the development of a portable, point-of-care platform for the practical monitoring of MP.

## Data Availability

The original contributions presented in the study are included in the article/Supplementary Material; further inquiries can be directed to the corresponding authors.
