# Supplementary material for: N,S-doped carbon dots derived from tryptophan for sensitive and selective detection of meropenem in live cells, human urine, and water samples
Source: Front Chem. 2026 Oct 27;14:1924756. doi: 10.3389/fchem.2026.1924756 (PMC13749302; doi:10.3389/fchem.2026.1924756)
Supplement: Supplementary file 1 [file DataSheet1.pdf]

Supporting Information

Materials

L-Tryptophan and thiourea were obtained from Sigma Aldrich, China for N,S-CDs preparation. The pharmaceutical-grade antibiotic MP was used for this. Other similar antibiotics which are levofloxacin, tetracycline, doxycycline, doripenem, ampicillin, imipenem, ertapenem, amoxicillin, ciprofloxacin, chloramphenicol, gentamicin and vancomycin were obtained from Aladdin, China. Accustandard USA supplied the common metal ions and anions. Sigma-Aldrich supplied glycine (Gly), alanine (Ala), cysteine (Cys), lysine (Lys), histidine (His), arginine (Arg), glutamic acid (Glu), and aspartic acid (Asp). We purchased glucose, fructose, sucrose, and lactose, among other common sugars from Energy Chemical Company, China. The required biochemicals for cell culture, including streptomycin, Dulbecco's phosphate buffered saline (DPBS), fetal bovine serum (FBS), Roswell Park Memorial Institute (RPMI) medium 1640, penicillin, and HEPES buffer solution was bought from Hyclone (USA). For the cytotoxicity measurements, the MTT (4,5-dimethylthiazol-2-yl)-2,5-diphenyltetrazolium bromide was obtained from Beyotime, China. All other reagents unless stated here were used as of analytical grade and used as such without further purifications. The deionized water (DI) was processed in our laboratory purification system and used throughout experiments.

Instrumentation

A UV-visible spectrophotometer (Cary 8454, Agilent Technologies, USA) was used to measure the absorption spectra of N,S-CDs and their interaction with MP. A Cary Eclipse fluorescence spectrophotometer (Agilent Technologies, USA) was used to measure the fluorescence emission and excitation spectra. Using KBr pellets and a Thermo Nicolet 380 FT-IR spectrophotometer (Thermo Fisher Scientific, USA), was used to acquire the FTIR spectra.

High resolution transmission electron microscopy (HR-TEM) images from a JEM advanced analytical electron microscope were used to examine the morphology and size distribution of the N,S-CDs. Powder X-ray diffraction (PXRD) patterns were recorded in order to investigate the crystalline structure of the N,S-CDs. A pH meter (HI 2210, Hanna Instruments, Woonsocket, RI, USA) was used to adjust the pH of each solution. The BioTek ELX800 microplate reader (Fisher Scientific, USA) was used to conduct the cytotoxicity (MTT) assay. The Leica TCS SP 5 II confocal laser scanning microscope (Leica Microsystems, USA) with a 63× oil immersion objective lens was used to take pictures of live cell fluorescence.

Cell Culture, Cytotoxicity and Live Cell Imaging

Human prostate cancer PC3 cell lines were cultured in RPMI 1640 medium supplemented with 10% foetal bovine serum (FBS) and 100 mg/mL of penicillin and streptomycin, at 37°C in a humidified atmosphere with 5% CO_2. Cells were plated in 96-well plates for the cytotoxicity experiment and allowed to adhere overnight. N,S-CD solutions were prepared by dissolving different amounts of N,S-CD powder (0, 0.2, 0.5, 1, 3, 5 and 10 mg) in 1 mL of deionised water, giving concentrations of 0, 0.2, 0.5, 1, 3, 5 and 10 mg/mL. These solutions were added to the wells (n = 5 replicate wells per concentration) and incubated for 24 hours. Following incubation, the medium was aspirated, and the cells were gently rinsed with DPBS. Subsequently, 0.5 mg/mL MTT solution was introduced to each well and incubated for 4 hours at 37°C. Formed formazan crystals were solubilised in 0.1 mL DMSO, and the absorbance was quantified at 570 nm using a BioTek ELX800 microplate reader. Cell viability was assessed utilising the formula:

$$\text{Cell viability (\%)} = (\text{Absorbance of treated cells} / \text{Absorbance of control cells}) \times 100$$

PC3 cells were cultured in 35 mm glass-bottom dishes and incubated overnight for fluorescence bioimaging. The cells were subsequently treated with a 0.2 mg/mL N,S-CDs solution (10 and 30 μL) for 30 minutes, after which they were washed with DPBS to eliminate excess probe. Thereafter, varying doses of MP (1 and 3 μM) were applied to the N,S-CD-stained cells and incubated for 10 minutes. Fluorescence confocal pictures were obtained using a Leica TCS SP 5 II confocal laser scanning microscope, equipped with a 63× oil immersion objective lens and a diode laser as the excitation light source.

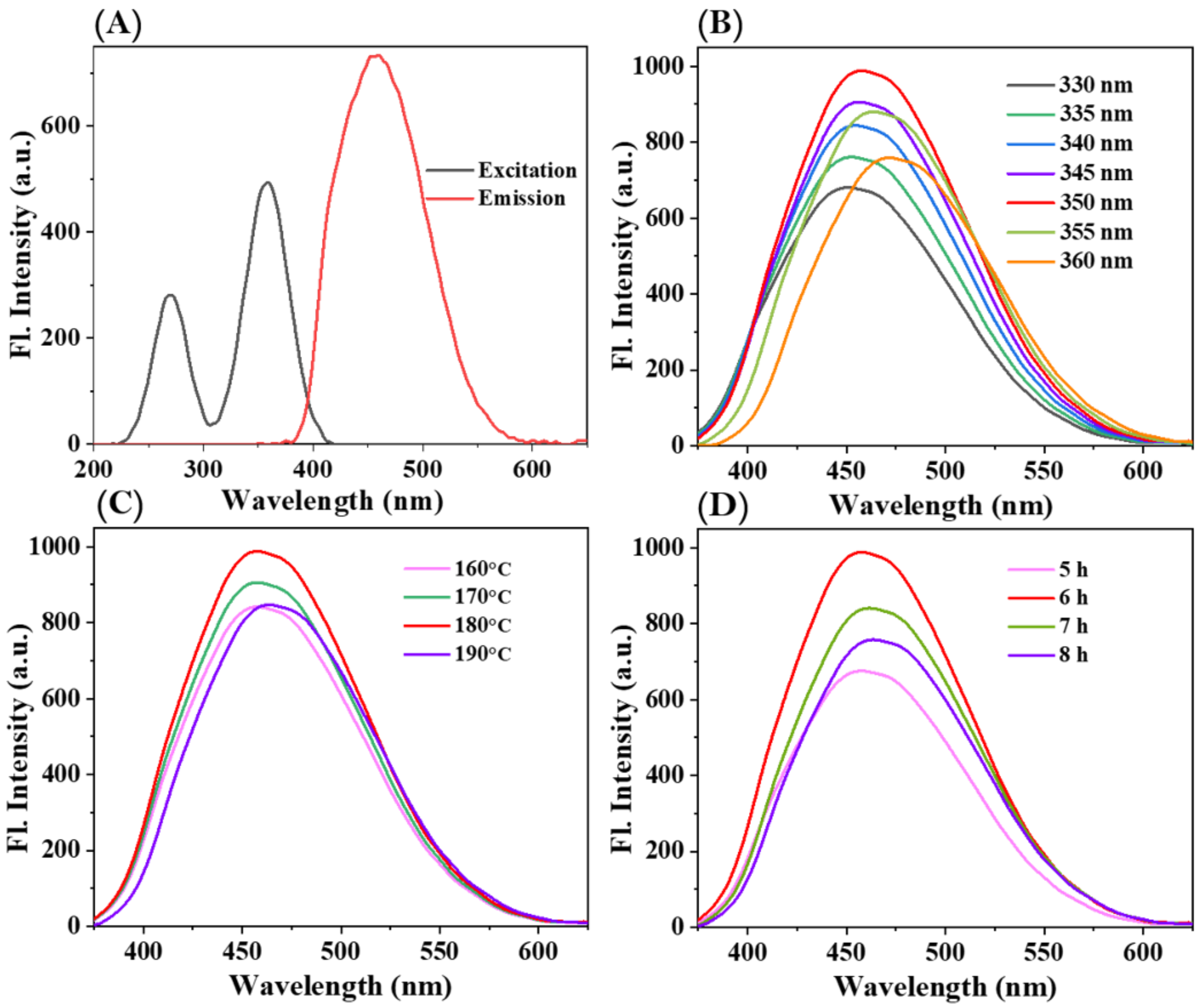

Fig. S1. (A) Excitation, emission spectra obtained for N,S-CDs, Fluorescence emission spectra obtained for optimization of reaction conditions for the preparation of N,S-CDs, (B) at different excitation wavelengths, (C) at different reaction temperatures and (D) at different reaction times.

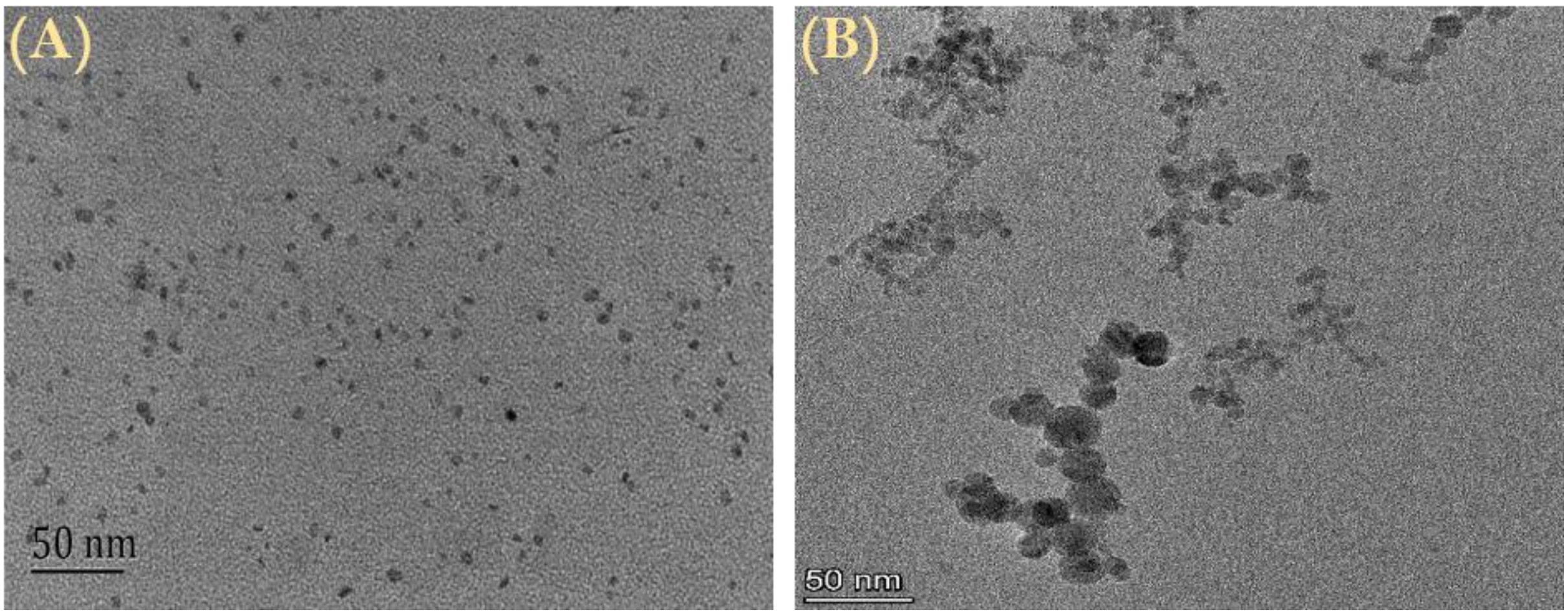

Fig. S2. TEM images obtained for N, S-CDs at different temperature synthesis (A) at 180° C and (B) at 190° C.

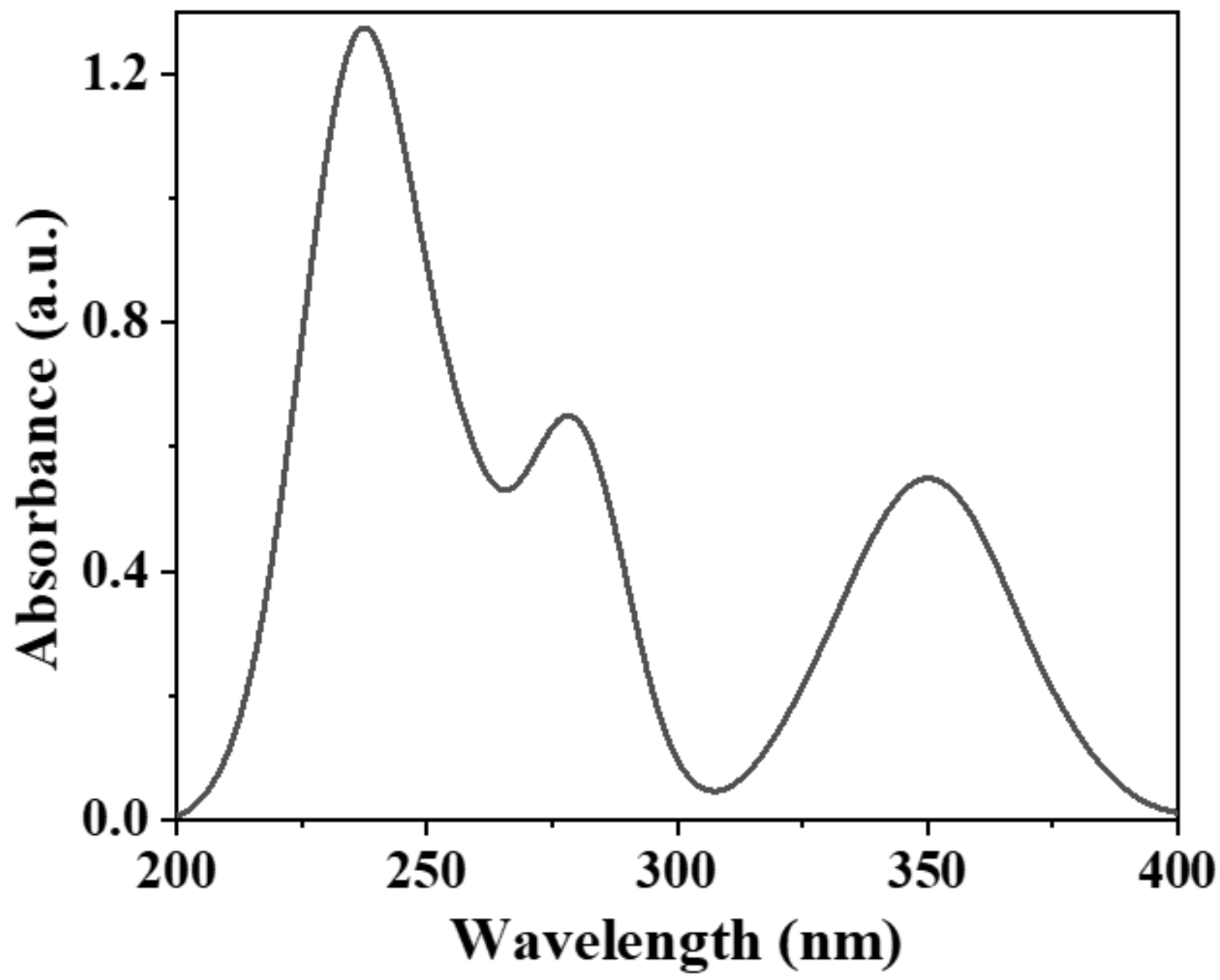

Fig. S3. UV-Vis absorbance spectrum obtained for N,S-CDs.

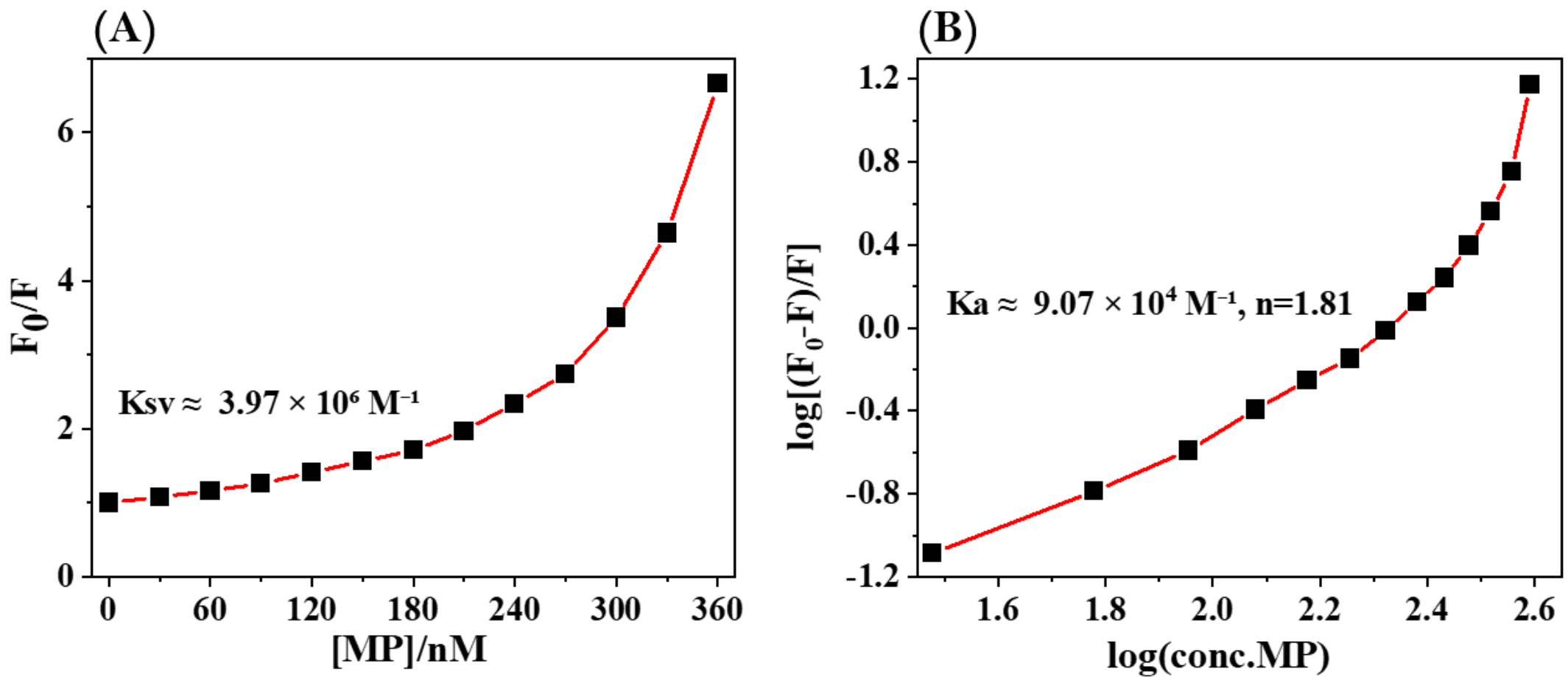

Fig. S4. (A) Stern-Volmer quenching plot and (B) binding constant plot obtained for N,S-CDs with different concentrations of MP (quencher).

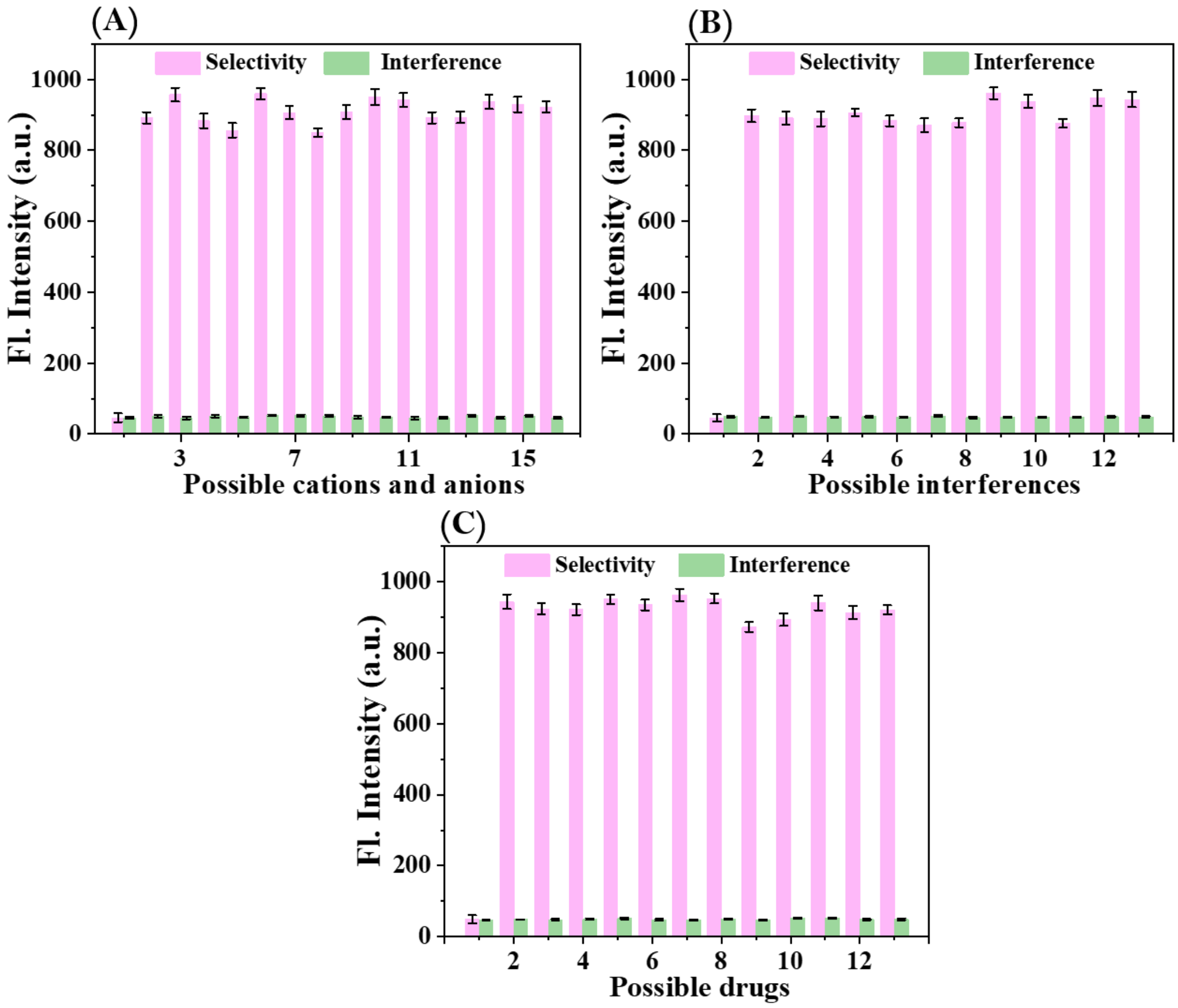

Fig. S5. Bar chart representation of selectivity and interference ability of N,S-CDs with possible interferences (A) possible cations and anions (1-16 are): MP, Cu^{2+}, Fe^{3+}, Pb^{2+}, Hg^{2+}, Cd^{2+}, Ca^{2+}, Mg^{2+} Zn^{2+}, Cl^-, Br^-, NO_3^-, NO_2^-, SO_4^{2-}, CH_3COO^-, SCN^-. (B) possible interfering biomolecules (1-13 are): MP, Gly, Ala, Cys, Lys, His, Arg, Glu, Asp, glucose, fructose, sucrose, and lactose. (C) possible similar drugs (1-12 are): MP, levofloxacin, tetracycline, doxycycline, doripenem, ampicillin, imipenem, ertapenem, amoxicillin, ciprofloxacin, chloramphenicol, gentamicin and vancomycin. For selectivity analysis 0.5 mM of each analyte was added separately to the probe solution, for interference analysis 0.5 mM of each analyte were added along with 0.4 µM of MP (n=3 times).

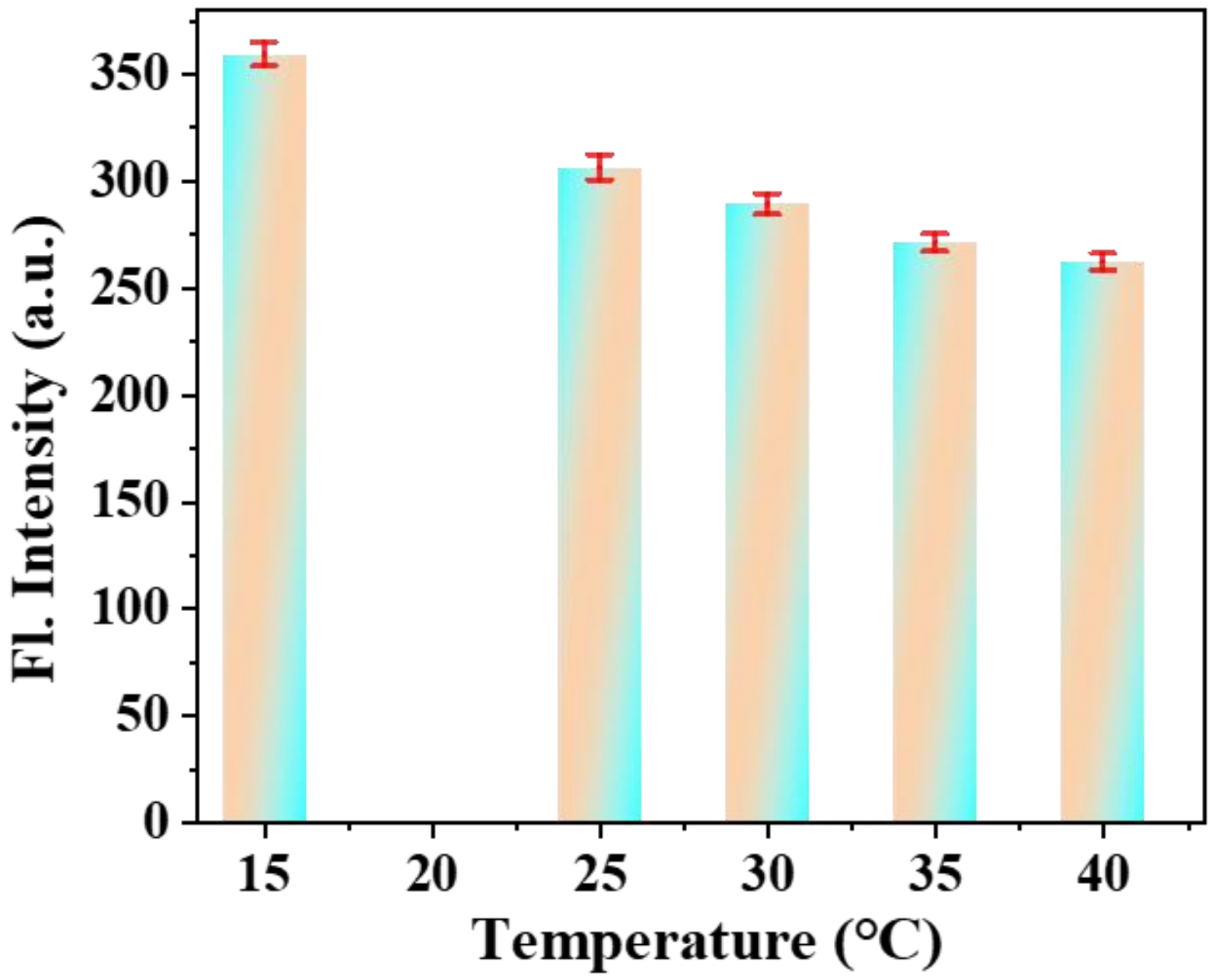

Fig. S6. Effect of temperature on the fluorescence intensity of the N,S-CDs with 0.4 μM of MP (15–40°C), showing a gradual decline in response with increasing temperature (n=3 times).

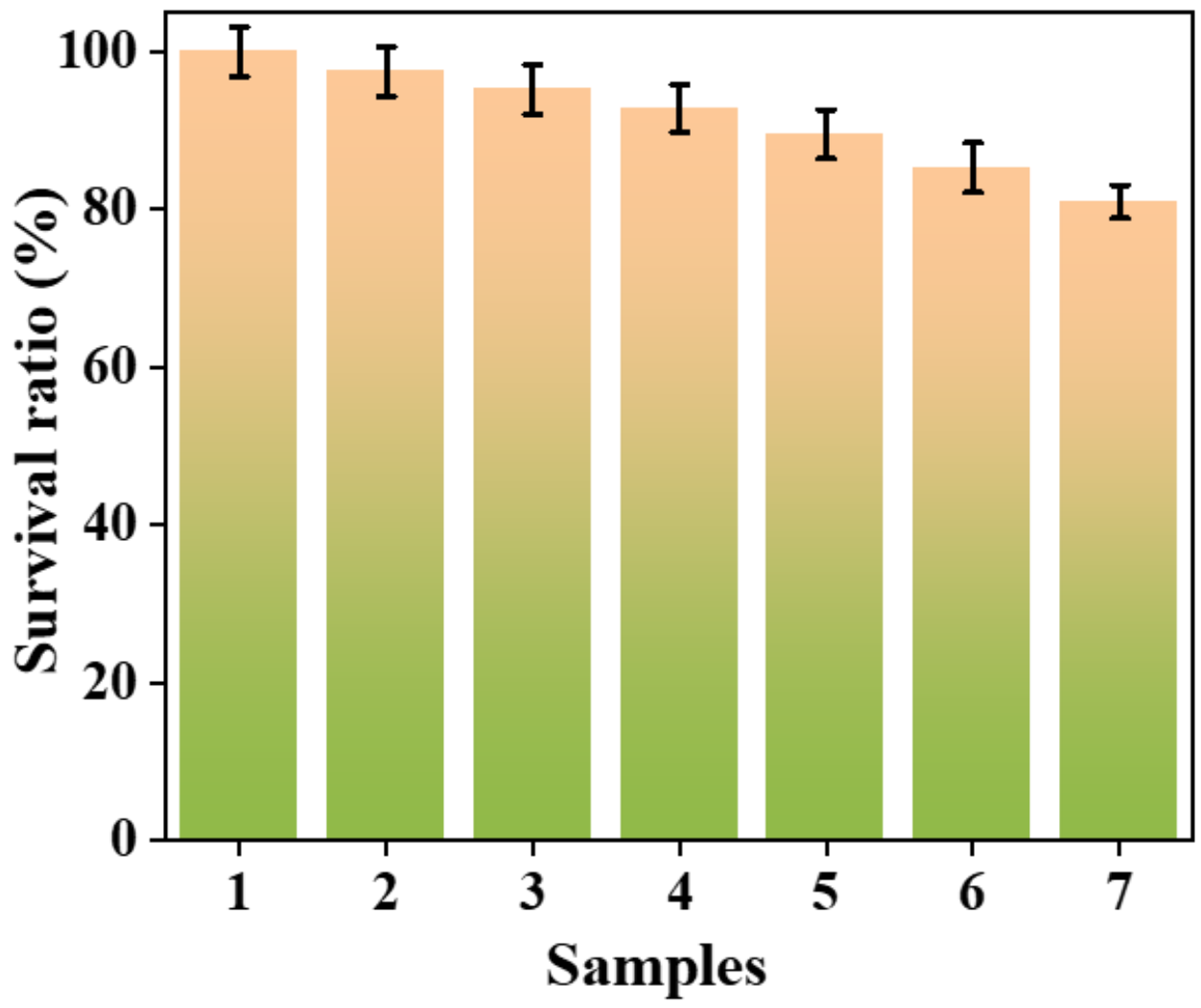

Fig. S7. Cell viability (MTT assay) of PC3 cells treated with different concentrations of N,S-CDs (0, 0.2, 0.5, 1, 3, 5 and 10 mg/mL) for 24 h. Data are presented as mean ± SD (n = 5).
